# Urinary retention as a postoperative complication associated with functional decline in elderly female patients with femoral neck and trochanteric fractures: A retrospective study of a patient cohort

**DOI:** 10.1097/MD.0000000000016023

**Published:** 2019-06-14

**Authors:** Toshihiro Higashikawa, Kenji Shigemoto, Kenichi Goshima, Daisuke Usuda, Masashi Okuro, Manabu Moriyama, Hiromi Inujima, Masahiro Hangyou, Kimiko Usuda, Shigeto Morimoto, Tadami Matsumoto, Shigeki Takashima, Tsugiyasu Kanda, Takeshi Sawaguchi

**Affiliations:** a Department of Geriatric Medicine, Kanazawa Medical University Himi Municipal Hospital, Kurakawa, Himi; b Department of Orthopedics and Joint Reconstructive Surgery, Toyama Municipal Hospital, Hokubumachi, Imaizumi, Toyama; c Kanazawa Medical University; d Department of Geriatric Medicine, Kanazawa Medical University, Uchinada, Kahoku-gun, Ishikawa; e Department of Urology, Kanazawa Medical University Himi Municipal Hospital, Kurakawa, Himi; f Toyama Municipal Hospital, Hokubumachi, Imaizumi, Toyama; g Department of Orthopedic Medicine, Kanazawa Medical University, Uchinada, Kahoku-gun, Ishikawa, Japan.

**Keywords:** activities of daily living, albumin, cognitive impairment, elderly, hip fractures, urinary retention

## Abstract

Urinary retention (UR) has been recognized as one of the most common postoperative complications after hip surgery in elderly. The objective of the present study was to evaluate risk for postoperative complications of UR in elderly female patients with femoral neck fractures.

We recruited 221 female patients (age 85.3 ± 7.0 years) with a history of hip surgery carried out at Toyama Municipal Hospital. UR occurred in 34 out of 221 cases (15.4%). Multiple logistic regression analysis was conducted to investigate the risk factors for UR, including age, body mass index (BMI), serum albumin, cognitive impairment, and activities of daily living (ADL).

The results showed significant association of UR with cognitive impairment (*P* = .005, odds ratio [OR] 4.11, 95% confidence interval [CI] 1.53–11.03), and ADL (*P* = .029, OR 2.61, 95% CI 1.11–6.18), under adjustment with age and BMI.

This study demonstrated that cognitive function and ADL were the important risk factors for UR, suggested that the postoperative management of UR is important with taking account of neurofunctional assistance and nursing care in daily living, especially in elderly female patients receiving surgery of femoral neck and trochanteric fractures.

## 1 Introduction

Surgery for hip fracture carries a significant risk of death with 7% dying in hospital.^[1]^ Lower extremity fractures below the hip region are a major, yet understudied, cause of morbidity and disability for geriatric patients.

Toyama city locates in the middle district in Toyama Prefecture which has an area of approximately 1200 square kilometers and has about 400,000 residents.^[2]^ The proportion of elderly people aged 65 and over to all the residents in Toyama city is over 30%. Toyama Municipal Hospital is a core hospital in Toyama Prefecture which has approximately 600 beds. In the hospital, an Elderly Bone Femoral Neck and Trochanteric Fracture Center has been installed with advanced technology and dedicated staffs to conduct surgery in patients with acute phase femoral neck and trochanteric fracture.

The elderly patients (>65 years old) which occupies the most part of femoral neck and trochanteric fracture also have concurrent medical diseases, thus not only the fracture but also whole-body control has been required. Among various diseases, femoral neck as well as trochanteric fracture are the major diseases which the nursing was required.^[3]^ Waiting time is also known to be a risk factor of postoperative complications and mortality of in patients underwent hip surgery.^[4,5]^ It is also known that the waiting time affects not only on the delay in the operation standby hour but also on the delay of the rehabilitation.^[6]^ Co-management of hip fracture patients by orthopedic surgeons and geriatricians is suggested to be effective to reduce length of hospital stay without negatively affecting major patient outcomes.^[7,8]^ On this account, multidisciplinary cooperation could be effective as conducted in Toyama Municipal Hospital. On the other hand, not only the operative delay but also postoperative morbidity has been crucial for prognosis of patients with orthopedic trauma.^[9]^ Regarding the postoperative complications, deep vein thrombosis, urinary retention, and pneumonia were found to be the most frequent complications observed in our hospital during 2016 to 2017. Previous studies have also suggested that the urinary retention is the major complications especially in the elderly.^[10–13]^ The risk factors of these complication have been under investigation. Several reports have suggested that malnutrition,^[14]^ activities of daily living (ADL),^[15]^ and cognitive function^[16]^ are suggested to be risk factors relevant to these complications. Regarding the sex difference, it is necessary to compare patients after adjusting for the prostate size of each of the groups in the case of male patients. Thus, the subjects in the present study confined to female patients to omit the bias related to the prostate size.

The present study was undertaken to investigate risk factors associated with major postoperative complications, urinary retention, in female elderly patients with postoperative surgery of femoral neck and trochanteric fractures.

## 2 Materials and methods

This retrospective study was carried out under approval (2018-06) of the Clinical Research Ethics Committee of Toyama Municipal Hospital. Written informed consent was obtained from all patients. The inclusion criteria were that patients were female with age of ≥65 years, who underwent surgery of femoral neck and trochanteric fracture in our institute. The exclusion criteria were the patients with an indication of conservative treatment, multiple trauma, pathologic fracture, in-hospital falls, transferred the hospital, or patients with no measurement of serum albumin. Demographic data were collected by physicians, specialist nurses, pharmacists, and medical affairs. The existence of the urinary retention was also examined in the same division. Body mass index (BMI) was calculated as weight (kg) divided by the square of height (m^2^). Waiting time (day) from hospitalization to entering the operating room was also calculated on each case.

In all cases, urethral catheter was inserted preoperatively according to a urinary retention manual originally created in our hospital. The method has been conducted in a consolidated manner, regardless of the subjectivity of physicians.

The urinary retention was defined as a state of difficulty in micturition under storage of 400 to 500 mL of urine in a bladder after removal of the catheter, symptoms of dysuria or decreases in micturition desire under >100 mL of residual urine. The cases were defined as positive of urinary retention (UR positive) and others were defined as negative of urinary retention (UR negative).

Serum albumin concentration was measured as a standard test package of blood tests at admission, and the value itself was used for the following statistical analysis as continuous variables. In addition, the data were divided into high albumin (≥3.5 g/dL) and low albumin (<3.5 g/dL) groups that were used for the following analysis as dichotomous variables.

Cognitive disorders were diagnosed by neuropsychiatrists based on the current Diagnostic and Statistical Manual of Mental Disorders (DSM-V). Degree of independence in everyday life on each case was assessed by a conventional ADL scale, which is classified by the 4 groups (J, A, B, C). The group J is mostly assistance-free, the group A is semi-bedridden mostly active during daytime, the group B is bedridden requiring assistance for daily activities, and the group C is bedridden requires assistance in bed.^[17]^ We divided them into 2 groups, J A (assistance non-required), and B C (assistance-required) group.

Data of history of hypertension, diabetes mellitus, circulatory disease, respiratory disease, renal disease, bone fracture and osteoporosis were collected from medical records of the cases.

The present study consists of continuous data, such as age, BMI, waiting time, serum albumin concentration, which were statistically evaluated by mean, standard deviation and Student *t* test.

The present study also consists of dichotomous data, such as categorized serum albumin concentration, cognitive disorder, ADL, and history of diseases, which were statistically evaluated by chi-square test.

Logistic regression analyses were performed using dichotomous UR positive and UR negative as dependent variables, and age, BMI, serum albumin, cognitive disorder, and ADL as independent variables.

All statistical analyses were performed with EZR (Saitama Medical Center, Jichi Medical University, Saitama, Japan).^[18]^

## 3 Results

**Figure 1:**
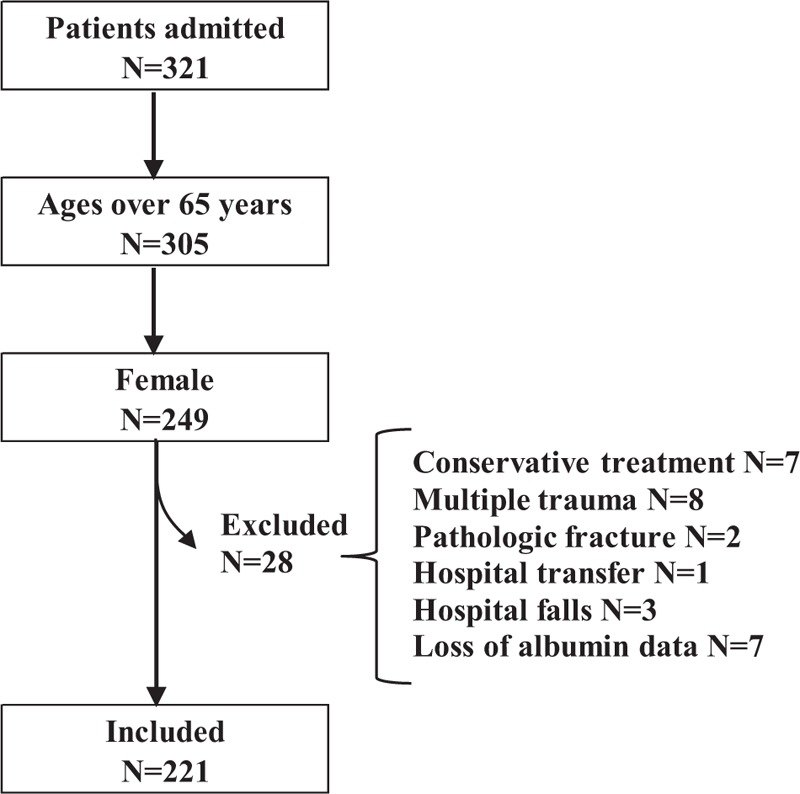
Flow chart of the study.

Initially 321 patients were admitted to the Toyama Municipal Hospital from January 1, 2016, to December 31, 2017, excluding 16 patients younger than 65 years, 7 patients with conservative treatment, 8 patients with multiple trauma, 2 patients with pathologic fracture, 1 patient with hospital transfer, 3 patients with in-hospital falls, and 7 patients with no measurement of serum albumin. Finally, 221 female patients were used as an analysis set of the present study, as depicted in Fig. 1. Among these, 8 patients were found urinary infection but were not excluded in this study.

Table 1 shows patient characteristics at the enrollment in this study. The patient population (N = 221) were divided into UR negative (N = 187) and UR positive (N = 34) groups. Age, BMI, waiting time, history of hypertension, history of diabetes mellitus, history of circulatory disease, history of respiratory disease, history of renal disease, history of bone fracture, and history of osteoporosis showed no significant differences between the groups. On the other hand, serum albumin, categorized serum albumin, cognitive impairment, and ADL showed significant differences between the groups (*P* = .006, .003, <.001, and <.001, respectively).

**Table 1:**
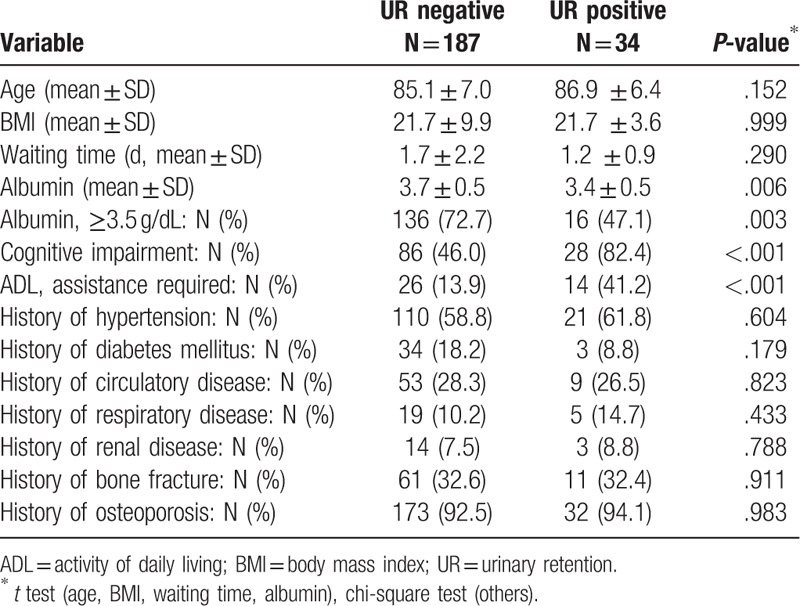
Patient characterictics at baseline.

Table 2 shows the multiple logistic regression analysis indicating that cognitive impairment (*P* = .005, odds ratio [OR] 4.11, 95% confidence interval [CI] 1.53–11.03) as well as ADL (*P* = .029, OR 2.61, 95% CI 1.11–6.18) were associated with the occurrence of UR under adjustment of age, sex, and BMI as covariates. Serum albumin did not significantly but marginally (*P* = .057) correlate with the occurrence of UR.

**Table 2:**
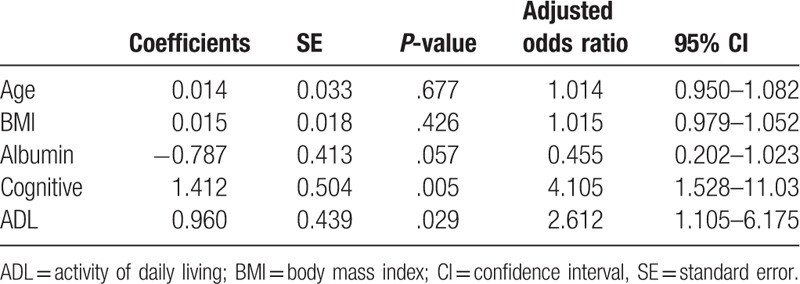
Results of logistic regression analysis.

**Figure 2:**
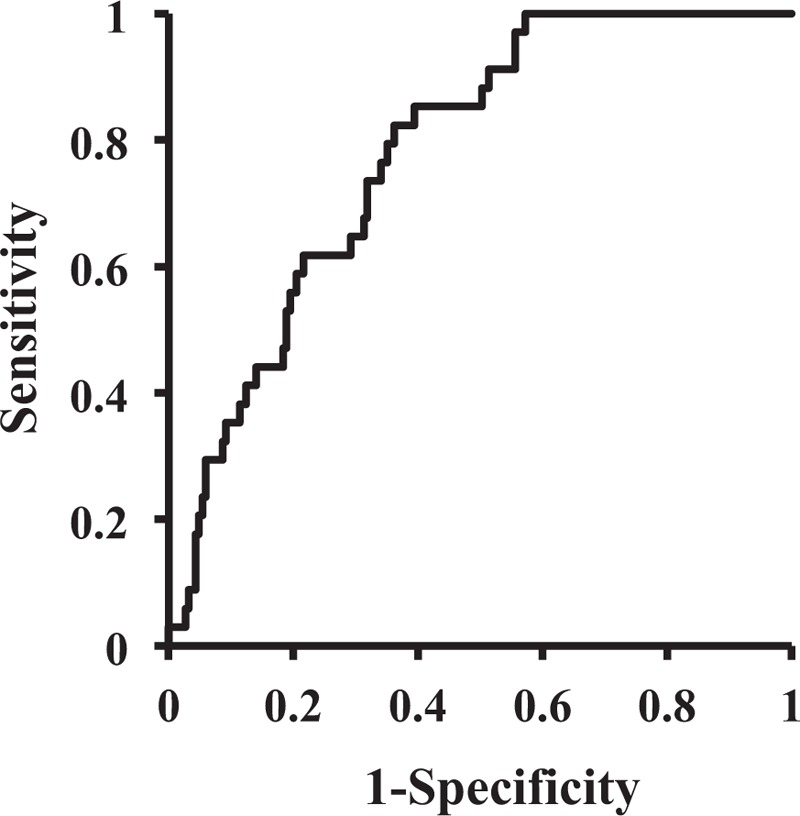
ROC curves of the logistic regression model. ROC = receiver operating characteristic.

Figure 2 shows the receiver operating characteristic (ROC) curve of the logistic model, UR positive and UR negative as dichotomous dependent variables, and age, sex, BMI, albumin, cognitive disorder, and ADL as independent variables. The area under the curve and its 95% confidence interval were 0.778 (0.707–0.850).

## 4 Discussion

In parallel with the advances in orthopedics, the number of elderly people who undergo surgery increases. Recently, the number of patients with femoral neck fracture has increased in Japan.^[19]^ In such situation, management of postoperative complications lead to better postoperative prognosis, and investigation of risk factors underlying such complications are important in this viewpoint. The present retrospective study has suggested that the serum albumin, cognitive impairment as well as ADL could be risk factors for UR in patients receiving postoperative surgery of femoral neck and trochanteric fractures.

Regarding the patient background, as shown in Table 1, serum albumin, categorized serum albumin, cognitive impairment, and ADL showed significant differences between the UR positive and UR negative groups (*P* = .006, .003, <.001, and <.001, respectively). The results suggested that serum albumin, cognitive impairment, and ADL were associated with UR occurrence. On the other hand, no significant differences were observed in the history of hypertension, history of diabetes mellitus, history of circulatory disease, history of respiratory disease, history of renal disease, history of bone fracture, and history of osteoporosis, indicating that the UR occurrence is less associated with history of such diseases.

The results of the multiple logistic regression analyses were shown in Table 2, which suggested that cognitive impairment (*P* = .005, OR: 4.11, 95% CI: 1.53–11.03) as well as ADL (*P* = .029, OR: 2.61, 95% CI: 1.11–6.18) were associated with the occurrence of UR under adjustment of age and BMI as covariates, indicating that cognitive impairment as well as ADL are variables independently associated with occurrence of UR. The validity of the model was assessed by ROC curve as shown in Fig. 2, which suggests that model was valid as it shows enough AUC with narrow confidence interval, that is, 0.778 (0.707–0.850). The association between serum albumin and UR occurrence was not significant but marginally significant (*P* = .057). Together with the result of significant difference between them as shown in Table 1, the serum albumin can play some role in the occurrence of UR. Cialic et al,^[12]^ also investigated elderly female patients with postoperative UR that serum albumin is significantly lower in post operative urinary retention (POUR) patients compared with NON-POUR patients. They concluded that although the level of albumin was significantly lower in the postoperative UR group, the magnitude of the difference was subtle and clinically no significance.

Regarding association of UR with cognitive disorders, Tobu et al,^[11]^ also investigated their association using logistic regression analysis and found association of UR with preoperative cognitive disorder and/or delirium. They suggested that preoperative cognitive disorder and/or delirium can lead to too early decannulation and the subsequent postoperative UR. Medication of anticholinergic agent in elderly patients is also known to be a risk factor for UR.^[20]^ The present study also found association of UR with ADL. Catheter drainage after acute urinary retention is known to have an impact on ADL and morbidity.^[15]^ Permanent indwelling urethral catheters can also induce deterioration of activities of daily living and stone formation.^[21]^ Early removal of the urethral catheter is known to have significant correlations with postoperative UR.^[11]^ These results have suggested that urinary retention could be due to catheterization which could also leads to deterioration of ADL. It is also suggested that cognitive impairment is associated with ADL in elderly patients with hip fracture.^[22]^ In general, if patients who decreased ADL occurred UR, the period of hospitalization tends to extend in parallel with indwelling catheterization. Therefore, as suggested in the present study the prediction of UR could be important especially in low ADL patients. The results of the present study could contribute such cases as a prediction tool. In any cases, managing of ADL as well as cognitive disorder could be important for better prognosis of postoperative hip fracture in elderly patients.

At the start of the study we also speculated association of UR with waiting time, but no association was found (UR negative: 1.7 ± 2.2 days, UR positive: 1.2 ± 0.9 days, *P* = .290). It is known that early surgery was not associated with improved function or mortality, but it was associated with reduced pain and length of stay and probably major complications among patients medically stable at admission.^[23]^ The average waiting time in Japan is approximately 4.2 days,^[4]^ but in Toyama Municipal Hospital the time is approximately 1.58 days. Although irrespective of UR occurrence the shortening of the waiting time can be important for the prognosis of patients after hip surgery.

The current study has several limitations. First, the present results have no control groups such that the patients whose waiting time to the surgery was extended, which interferes investigating causal association. This should be taken into consideration on the interpretation of the present results. Second, the present study population was all female patients, but UR is also a serious complication for men. Risk factors for postoperative urinary retention in men undergoing hip surgery has also been studied, which suggests that spinal anesthesia and age older than 70 years are the risk factors.^[24]^ Third, other than investigated in the present study, there could be many risk factors for UR that should be taken into consideration, such as cortisol, hemoglobin, folic acid, thyroid stimulating hormone, opioid use, preadmission bladder function, etc., are the factors relevant to postoperative UR.^[12]^ Spinal and opiate anesthesia have known to induce urinary retention,^[25,26]^ thus the anesthetics can also be a factor to induce UR in the patients in the present study. Therefore, further investigation will be required to comprehensively evaluate risk of UR and to elucidate key factors relevant to the prognosis of postoperative femoral fracture.

## 5 Conclusion

In conclusion, we retrospectively evaluated the risk factors for UR in patients with femoral neck and trochanteric fractures, showed that cognitive impairment and ADL were the important risk factors for UR. Postoperative management of UR could be important with taking account of neurofunctional assistance and nursing care in daily living, especially in elderly female patients receiving surgery of femoral neck and trochanteric fractures.

## Author contributions

**Conceptualization:** Kimiko Usuda.

**Data curation:** Kenji Shigemoto, Kenichi Goshima, Hiromi Inujima.

**Formal analysis:** Toshihiro Higashikawa, Kenji Shigemoto, Kenichi Goshima, Masahiro Hangyou, Kimiko Usuda.

**Investigation:** Toshihiro Higashikawa, Daisuke Usuda, Manabu Moriyama, Kimiko Usuda.

**Methodology:** Daisuke Usuda, Masahiro Hangyou.

**Supervision:** Masashi Okuro, Manabu Moriyama, Shigeto Morimoto, Tadami Matsumoto, Shigeki Takashima, Tsugiyasu Kanda, Takeshi Sawaguchi.

**Writing – original draft:** Toshihiro Higashikawa.

**Writing – review & editing:** Toshihiro Higashikawa.

Toshihiro Higashikawa orcid: 0000-0001-8690-5473.
